# Incomplete Faces Do but Masked Faces Do Not Affect Mind Perception

**DOI:** 10.1177/00332941241269547

**Published:** 2024-07-30

**Authors:** Farid Pazhoohi, Keina Aoki, Alan Kingstone

**Affiliations:** School of Psychology, 6633 University of Plymouth, Plymouth, UK; Department of Psychology, 8166 University of British Columbia, Vancouver, BC, Canada

**Keywords:** Face mask, mind perception, mental attribution, face recognition

## Abstract

The human face plays a critical role in how we perceive the minds of others. The current research across two studies explored whether face masks also impact mind perception, with the expectation that they lead to lower attributions of agency and experience to individuals, making them seem less mentally capable due to their association with reduced facial expression perception and impaired communication. In the first study, participants’ ratings of masked and unmasked faces for agency and experience did not yield significant differences, suggesting that wearing a face mask does not affect the perception of the mind. To explore whether these findings applied when the lower face was cropped instead of masked, results of the second study showed that removing the lower face led to decreased agency ratings, but similar to the first study, there were no changes in experience ratings. Altogether, our results showed that wearing face masks does not reduce the perception of mental capacity. Moreover, female faces received higher ratings for both agency and experience compared to male faces. The complex relationship between face masks, gender, and mind perception warrants further exploration.

The perception of the human face is a complex cognitive ability that plays a pivotal role in our daily social interactions. Individuals tend to attribute mind to subjects and/or objects that have features resembling those of human faces (for a review see Deska & Hugenberg, 2017), with eyes in particular playing a crucial role (Khalid et al., 2016; Looser & Wheatley, 2010). Previous research has shown that a disruption in configuration processing of faces, including face inversion, affects the attribution of mental states to faces (Hugenberg et al., 2016; Krumhuber et al., 2019). Moreover, mental attributions are influenced by perception of emotions from faces; for example, individuals attribute more mind to faces expressing happy emotions compared to neutral faces (Bowling & Banissy, 2017; Krumhuber et al., 2019).

In the aftermath of the recent coronavirus pandemic (COVID-19), a myriad of research on verbal communication has shown that language processing and speech comprehension are negatively affected when faces are masked (Giovanelli et al., 2021; Saunders et al., 2021; cf. Bourke et al., 2023). Face masks negatively affect interconnectedness with the speaker, reduce hearing ability, and speech understanding (Saunders et al., 2021), as well as affecting one’s perception of their own and other’s speech intelligibility (Ribeiro et al., 2022; cf. Cohn et al., 2021; for a review see Badh & Knowles, 2023).

Recent work on facial expression recognition has also shown that face masks, by obscuring facial features, diminish accurate identification and perception of emotions, and decrease confidence in emotion perception (e.g., Carbon, 2020; Fitousi et al., 2021; Gori et al., 2021; Grahlow et al., 2022; Grenville & Dwyer, 2022; Grundmann et al., 2021; McCrackin et al., 2022; Pazhoohi et al., 2021). Such effects appear to be accentuated in individuals with autistic traits, who have difficulties with social interactions (e.g., Pazhoohi et al., 2021; Tate et al., 2023). In addition to reduction in emotion perception and recognition, research has shown that wearing face mask negatively affects perceived empathy (Proverbio et al., 2023; Wong et al., 2013).

As face masks have been associated with reduced facial expression perception, impaired verbal and nonverbal communication, as well as decreased empathy, we predicted that masked faces would be perceived to have lower agency (the ability to do) and experience (the ability to feel). While it is clear that face masks impede the perception of facial expressions and verbal communication, the impact on mind perception is less straightforward. The current study aimed to test whether face masks affect mind perception (Gray et al., 2007). Mind perception, which encompasses attributions of mental capacities such as agency (the ability to do) and experience (the ability to feel), can be influenced by the available social cues. Masks obscure critical facial features that contribute to these social cues, potentially altering the perception of an individual’s mental state. The obscuration may lead observers to infer that a masked individual has diminished capacity for actions and emotions due to reduced access to the expressive information typically conveyed through the lower part of the face. Therefore, we predicted that masked faces might be perceived to have lower agency and experience, as the reduction in visible facial information could translate to a perceived reduction in mental presence and emotional expressiveness. Additionally, we hypothesized that men are more likely to be perceived as possessing higher levels of agency, reflecting societal stereotypes that associate males with action, decision-making, and control. Conversely, women are hypothesized to be perceived as having greater capacity for experience, aligning with traditional views that emphasize empathy, emotional sensitivity, and nurturing roles.

## Study 1

Study 1 investigated whether face masks affect mind perception, specifically agency and experience. In this study, we asked men and women to rate male and female faces, both with and without face masks, on perceptions of agency (the ability to act) and experience (the ability to feel).

### Methods

#### Participants

A G*Power analysis for a 2 × 2 × 2 mixed effects design indicated that 138 participants would be sufficient to detect a small effect size (*f* = 0.10, *β* = 0.80). Due to the system of participant recruitment at the University of British Columbia we ended up oversampling, and a total of 204 participants (48 men and 156 women), between the ages of 18 and 39 years (*M* = 20.81, *SD* = 2.92) were recruited. Students received course credit in return for their participation. Each gave written informed consent.

#### Stimuli and procedure

**Figure 1.**
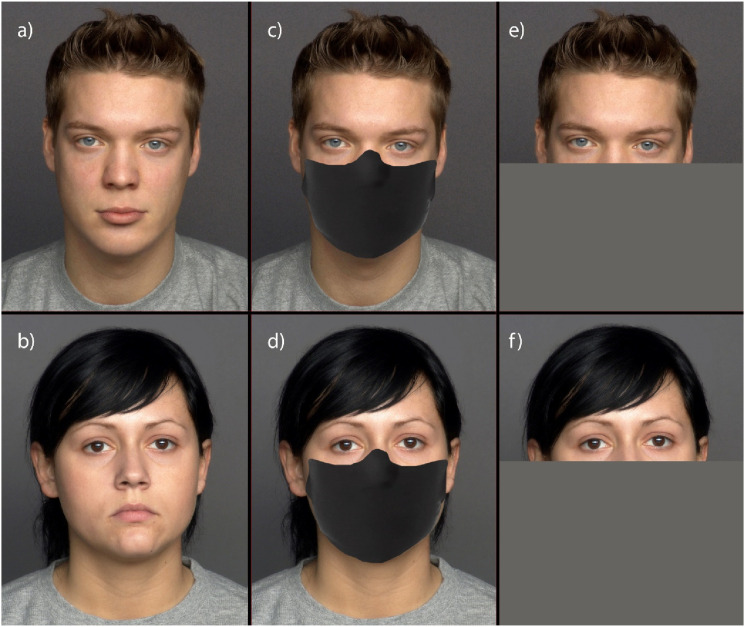
Examples of full (a and b), and upper half male and female faces (c and d) used in Study 1; examples of full (a and b) and upper half faces (e and f) used in Study 2.

Images of 10 male and 10 female faces with neutral expressions were obtained from the FACES database (Ebner et al., 2010). These 20 faces were aged between 19 and 31 years. Another set of 20 stimuli of the same identities were created by superimposing a facial mask on the original images. Each set of stimuli which included masked and unmasked faces were randomised and presented in separate blocks. After consenting to participate in the study, participants answered sociodemographic questions. This was a within-subjects experimental design and participants randomly observed either the block with facial masks first or the block with unmasked faces first (see Figure 1 for examples). Participants were asked to respond to two questions. The first question was “How would you rate the agency (ability to do) of this person?” and “How would you rate the experience (ability to feel) of this person?” on a slider. The slider allowed participants to drag a bar to indicate their ratings of agency and experience from 0 (not at all) to 100 (extremely). The slider was labelled in intervals of 10. Each question included a note to remind participants that agency “comes from the ability of these characters to possess: self-control, morality, memory, emotion recognition, planning, communication, and thought”; and that experience “comes from the ability of these characters to feel: hunger, pain, pleasure, rage, desire, personality, consciousness, pride, embarrassment, and joy”.

### Results

Table 1 shows descriptive results for agency and experience ratings. A linear mixed model was conducted to investigate the effect of Participant Sex, Stimulus Sex, and Mask (presence/absence) on the rating of agency, with Participants as a random effect. All post hoc comparisons throughout the results of this and the next study were performed using Bonferroni correction, and this is reflected in the *p* values. Results showed that there was a significant main effect for Participants Sex and Stimulus Sex. Women (*M* = 67.88, *SEM* = 1.11, 95% CI [65.70, 70.06]) provided higher agency ratings for stimuli than men (*M* = 64.61, *SEM* = 1.99, 95% CI [60.68, 68.54]; Table 2); and participants rated female stimuli (*M* = 66.55, *SEM* = 1.15, 95% CI [64.28, 68.82]) higher on agency than male stimuli (*M* = 65.95, *SEM* = 1.15, 95% CI [63.68, 68.22]). The main effect of face mask and its interactions were not significant (Table 2).

**Table 1.** Means and Standard Deviations of Agency and Experience Ratings as a Function of Participant Sex, Stimulus Sex, and Mask Condition.

	Agency		Experience	
	M	SD	M	SD
Participant sex				
Male	64.61	18.54	60.96	20.65
Female	67.88	18.52	65.20	21.11
Stimulus sex				
Male	66.55	18.69	61.63	21.40
Female	67.68	18.44	66.78	20.42
Mask				
Unmasked	67.16	18.48	64.31	20.96
Masked	67.07	18.68	64.09	21.19

**Table 2.** Estimates for the Effects of Facial Mask, Stimuli Sex, Participant Sex on the Ratings of Agency in Faces.

Effect	*β*	*SE*	*df*	*t*	*p*
(Intercept)	69.28	1.30	390.6	53.10	<.001***
Mask	0.40	0.45	7950	0.88	.377
Stimuli sex	2.52	1.01	7950	2.51	.012 *
Participant sex	5.49	2.69	390.6	2.04	.042 *
Mask × stimuli sex	0.62	0.64	7950	0.97	.332
Mask × participant sex	0.80	0.93	7950	0.87	.386
Stimuli sex × participant sex	2.16	2.07	7950	1.04	.299
Mask × stimuli sex × participant sex	0.10	1.31	7950	0.08	.940

*Note.* *p < .05, **p < .01, ***p < .001.

Another linear mixed model was conducted to investigate the effect of Participant Sex, Stimulus Sex, and Mask (presence/absence) on the rating of experience, with Participants as a random effect. Results showed a significant main effect for Stimulus Sex, with females being perceived to have more experience (the ability to feel) than males. This main effect was qualified by a two-way Stimuli Sex × Mask interaction (Table 3), with masks having no effect on the perception of experience for male faces (unmasked: *M* = 60.72, *SEM* = 1.33, 95% CI [58.10, 63.34]; masked: *M* = 60.74, *SEM* = 1.33, 95% CI [58.12, 63.36]), but they reduced the perceived experience for female faces (unmasked: *M* = 65.94, *SEM* = 1.33, 95% CI [63.32, 68.56] vs. masked: *M* = 64.92, *SEM* = 1.33, 95% CI [62.30, 67.54], *p* < .001).

**Table 3.** Estimates for the Effects of Facial Mask, Stimuli Sex, Participant Sex on the Ratings of Experience in Faces.

Effect	*β*	*SE*	*df*	*t*	*p*
(Intercept)	66.75	1.45	384.1	45.34	<.001***
Mask (unmasked)	0.82	0.50	7950	1.63	.104
Stimuli sex (male)	−2.99	1.12	7950	−2.66	.008 **
Participant sex (male)	−5.72	3.04	384.1	−1.88	.060
Mask × stimuli sex	−1.71	0.71	7950	−2.40	.016 *
Mask × participant sex	0.42	1.03	7950	0.41	.684
Stimuli sex × participant sex	−0.29	2.31	7950	−0.12	.901
Mask × stimuli sex × participant sex	1.32	1.46	7950	0.90	.367

*Note.* *p < .05, **p < .01, ***p < .001.

### Discussion

The results of the first study, where participants rated masked and unmasked faces for agency and experience, showed that female faces were rated as having more agency and experience than male faces. While masks did not affect the perception of agency, they did reduce the perception of experience, but only for female faces.

## Study 2

To investigate whether these effects may be specific to masks or will extend to the general removal of information from the lower half of the face, we conducted a second study. In this study, we cropped the portion of the face that had been covered in Study 1 and presented only the upper half of the face to the participants.

### Methods

#### Participants

A total of 194 participants (34 men and 160 women), between the ages of 18 and 31 years (*M* = 20.34, *SD* = 1.94), were recruited from the University of British Columbia, and received course credit in return for their participation.

#### Stimuli and procedure

The 20 full faces in Study 1 served as stimuli. Another set of 20 stimuli of the same identities were created by cropping the lower face of the stimuli (see Figure 1). The procedure was the same as Study 1.

### Results

Table 4 shows descriptive results for agency and experience ratings. As in Study 1, a linear mixed model was conducted to investigate the effect of Participant Sex, Stimuli Sex, and Face (half vs. full) on the ratings of agency, with Participants as a random effect. Results (see Table 5) showed a main effect of Face, with higher agency scores for full faces (*M* = 61.13, *SEM* = 1.32, 95% CI [58.52, 63.74]) than half faces (*M* = 59.71, *SEM* = 1.32, 95% CI [57.11, 62.32]). This main effect was qualified by Participant Sex, with males rating full faces (*M* = 60.93, *SEM* = 2.40, 95% CI [56.20, 65.67]) significantly higher on agency than half faces (*M* = 57.71, *SEM* = 2.40, 95% CI [52.97, 62.44], *p* < .001), whereas female participants considered them equivalent, (full faces: *M* = 61.32, *SEM* = 1.11, 95% CI [59.12, 63.47]; half faces: *M* = 60.93, *SEM* = 1.11, 95% CI [59.50, 63.87], *p* = .999).

**Table 4.** Means and Standard Deviations of Agency and Experience Ratings as a Function of Participant Sex, Stimulus Sex, and Face Condition.

	Agency		Experience	
	M	SD	M	SD
Participant sex				
Male	59.43	20.75	54.84	22.31
Female	61.49	20.35	61.25	21.52
Stimulus sex				
Male	60.13	20.66	63.06	21.84
Female	62.22	20.18	57.47	21.42
Face				
Half face	61.02	20.84	60.27	21.97
Full face	61.24	20.02	59.98	21.62

**Table 5.** Estimates for the Effects of Face (Half vs. Full), Stimuli Sex, Participant Sex on the Ratings of Agency.

Effect	*β*	*SE*	*df*	*t*	*p*
(Intercept)	61.98	1.15	234.11	54.06	<.001***
Face	2.14	0.55	7560	3.88	<.001***
Stimuli sex	1.32	0.54	7560	2.44	.015 *
Participant sex	3.19	2.74	234.11	1.17	.245
Face × stimuli sex	3.48	0.76	7560	4.57	<.001***
Face × participant sex	3.49	1.32	7560	2.65	.008 **
Stimuli sex × participant sex	5.62	1.29	7560	4.36	<.001***
Face × stimuli sex × participant sex	0.27	1.82	7560	0.15	.882

*Note.* *p < .05, **p < .01, ***p < .001.

There was also a significant main effect for Stimulus Sex, with female faces (*M* = 60.58, *SEM* = 1.32, 95% CI [57.97, 63.19]) being rated higher on agency than male faces (*M* = 60.26, *SEM* = 1.32, 95% CI [57.11, 62.32]). This main effect was qualified by an interaction with Participant sex, with males rating female faces (*M* = 58.07, *SEM* = 2.41, 95% CI [53.27, 62.89]) lower on agency than male faces (*M* = 60.50, *SEM* = 2.40, 95% CI [55.81, 65.28], *p* = .020). However, female participants rated male faces (*M* = 59.97, *SEM* = 1.11, 95% CI [57.82, 62.17]) lower on agency than female faces (*M* = 63.04, *SEM* = 1.11, 95% CI [60.92, 65.19], *p* < .001).

Finally, there was a Stimulus Sex × Face interaction, with the agency for female faces being equivalent for full (*M* = 60.40, *SEM* = 1.37, 95% CI [57.70, 63.09]) and half faces (*M* = 60.78, *SEM* = 1.36, 95% CI [58.10, 63.49], *p* = .999), whereas for male faces agency fell from full (*M* = 61.87, *SEM* = 1.35, 95% CI [59.20, 64.54], *p* < .001) to half faces (*M* = 58.65, *SEM* = 1.36, 95% CI [55.97, 61.33], *p* < .001).

In summary, men and women perceive agency in faces differently depending on the sex of the stimulus, with men perceiving less agency in female faces, while women perceive less agency in male faces. Men also see less agency in half faces than full faces, while women see them equivalently. Finally, regardless of participant sex, the agency of male full faces plummeted when they were halved, whereas female faces held steady across formats.

**Figure 2.**
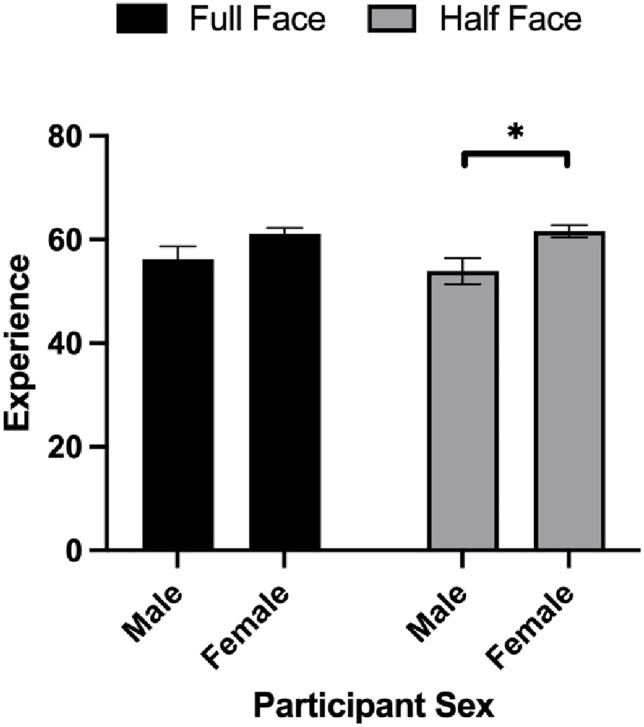
Experience ratings as a function of face (full vs. half) and participant sex. *p < .05.

Another linear mixed model was conducted to investigate the effect of Participant Sex, Stimuli Sex and Face (half vs. full) on the rating of experience, with Participants as a random effect. The results showed a significant main effect for Stimuli Sex and interaction for Participant Sex × Face (Table 6). For Stimuli Sex, the results showed that participants rated female faces (*M* = 61.10, *SEM* = 1.38, 95% CI [58.38, 63.83]) to have more experience compared to male faces (*M* = 55.29, *SEM* = 1.38, 95% CI [52.57, 58.00]). For the Participant Sex × Face interaction, women rated half faces (*M* = 61.64, *SEM* = 1.16, 95% CI [59.36, 63.92]) higher on experience than male participants (*M* = 53.86, *SEM* = 2.51, 95% CI [48.92, 58.80], *p* = .031). Meanwhile, male (*M* = 56.23, *SEM* = 2.51, 95% CI [51.24, 61.10]) and female participants *M* = 61.09, *SEM* = 1.16, 95% CI [58.77, 63.42], *p* = .124) rated full faces similarly (Figure 2).

**Table 6.** Estimates for the Effects of Face (Half vs. Full), Stimuli Sex, Participant Sex on the Ratings of Experience.

Effect	*β*	*SE*	*df*	*t*	*p*
(Intercept)	63.55	1.20	236.2	53.05	<.001***
Face	1.10	0.59	7560	1.87	.062
Stimuli sex	4.90	0.58	7560	8.51	<.001***
Participant sex	3.84	2.86	236.2	1.34	.181
Face × stimuli sex	1.13	0.81	7560	1.39	.164
Face × participant sex	4.34	1.41	7560	3.09	.002**
Stimuli sex × participant sex	2.18	1.38	7560	1.58	.113
Face × stimuli sex × participant sex	2.97	1.94	7560	1.53	.126

*Note.* *p < .05, **p < .01, ***p < .001.

### Discussion

The results of Study 2 showed that removing the lower face decreased ratings of agency. Moreover, each sex provided higher agency ratings for same-sex faces than opposite-sex ones; for example, men rated male faces higher on agency than female faces. However, both men and women rated female faces higher on experience than male faces.

### General Discussion

Across two studies, this research examined if mind perception is affected when viewing incomplete faces that are either concealed behind a face mask (Study 1) or missing lower facial features (Study 2). Our results from Study 1 showed that relative to full faces, a face mask does not influence agency and experience ratings of the perceived mind. This means that while previous research has shown that wearing a face mask disrupts emotion perception and identification (e.g., Carbon, 2020; Fitousi et al., 2021; Gori et al., 2021; Grahlow et al., 2022; Grenville & Dwyer, 2022; Grundmann et al., 2021; McCrackin et al., 2022; Pazhoohi et al., 2021), perceived empathy (e.g., Proverbio et al., 2023), and negatively affects speech comprehension (e.g., Giovanelli et al., 2021; Saunders et al., 2021), it does not make the targets appear less mentally capable. In other words, these studies primarily focused on the immediate perceptual and cognitive impacts of mask-wearing, such as difficulty in reading emotions and understanding speech, which are critical for social interactions. However, our study extends this line of research by examining a deeper cognitive aspect—mind perception, specifically the perception of agency and experience. The absence of an effect on agency and experience ratings suggests that while masks impede certain social and communicative processes, they do not make the targets appear less mentally capable.

The results of Study 2, in which the area of the face covered by the mask was cropped, showed that agency ratings decreased, but experience ratings did not, when compared to full faces. This suggests that incomplete faces, where the information from the lower face is missing, affect the perception of agency (the ability to do) but not affect the experience domain (ability to feel) of mind perception. This difference between the studies suggests that the physical presence of a mask might uniquely influence mind perception compared to simply missing facial features. The decrease in agency ratings in Study 2 aligns with findings in face perception literature, indicating that certain facial features are critical for conveying specific mental states and capabilities.

Our results dovetails with the argument that empathizing (understanding and sharing the feelings of others) and mentalizing (understanding others’ mental states) abilities are distinct and rely of different neural circuits (Singer, 2006). This distinction is further supported by the neuroanatomical separation of emotion processing and mentalizing abilities (Mitchell & Phillips, 2015).

Moreover, our findings regarding gender differences in mind perception revealed that individuals attributed higher agency and experience to female faces than to male faces. This contrasts with some research on objectification and mind perception, which has shown that individuals often attribute less agency to women (e.g., Loughnan et al., 2010; Vaes et al., 2011). However, women are generally considered warmer than men (e.g., Borau et al., 2021; Ebert et al., 2014), a trait associated with humanness (Au et al., 2021; Fiske et al., 2007). Additionally, attractiveness, which influences mind perception (Alaei et al., 2022), may play a role, as women are often perceived as more attractive than men (e.g., Palumbo et al., 2017). The interplay between face masks, attractiveness, gender, and mind perception warrants further investigation.

One limitation of the current study is the lack of information on participants’ ethnicity or cultural background. Research experiments on social cognition tend to be influenced by cultural and ethnic diversity, which can significantly impact the generalizability and applicability of the findings. Future studies should consider including this information to provide a more comprehensive understanding of the social cognitive processes across diverse populations. Another limitation of the is the use of a database consisting solely of Western European faces as our stimuli. This choice restricts the diversity of facial features represented in the study and may not accurately reflect the wide range of facial characteristics found in other ethnic and cultural groups. Future research should aim to incorporate a more diverse set of stimuli, including faces from various ethnicities and cultural backgrounds, to ensure broader applicability and inclusivity of the results. Moreover, the disproportionately high number of female participants compared to male participants is another limitation of the current research. This discrepancy mirrors the enrollment ratios in the psychology program at the University of British Columbia, where females are overrepresented. Therefore, while the study provides valuable insights, future research should aim for a more balanced sample to better understand and accurately reflect the nuances of sex differences in mind perception.

In conclusion, the current research across two studies examined how incomplete faces, due to face masks and missing lower facial features, affect our perception of others’ minds. The results showed that wearing a face mask does not diminish the perception of mental capability, suggesting that essential cognitive attributions remain intact despite partial facial occlusion. However, incomplete faces, such as those with missing lower facial features, do impact the perception of one’s ability to do (agency), as well as reducing the perception of ability to feel (experience) in female, but not male, faces. The latter indicates a gender-specific response to facial incompleteness. Additionally, female faces were generally attributed higher agency and experience ratings than male faces, highlighting a potential bias in mind perception based on gender. These findings suggest a complex interaction between facial completeness, gender, and the perception of mental capabilities. The differential impact on female and male faces underscores the need to explore how cultural and societal norms influence these perceptions. Moreover, the role of attractiveness in moderating these effects warrants investigation to understand its contribution fully. Previous research has indicated face mask and incomplete faces affect perception of attractiveness (Pazhoohi & Kingstone, 2022). Future research should aim to disentangle these variables further and consider additional factors such as ethnicity, age, and context to provide a more comprehensive understanding of how face masks and incomplete facial features influence social cognition. This knowledge is particularly relevant in contemporary settings where face masks have become commonplace due to global health concerns, emphasizing the importance of maintaining effective social interactions in the presence of facial occlusions.

## Data Availability

Data sharing not applicable to this article as no datasets were generated or analyzed during the current study.
